# The mitochondrial gene *orfH79 *plays a critical role in impairing both male gametophyte development and root growth in CMS-Honglian rice

**DOI:** 10.1186/1471-2229-10-125

**Published:** 2010-06-24

**Authors:** Xiaojue Peng, Kun Wang, Chaofeng Hu, Youlin Zhu, Ting Wang, Jing Yang, Jiping Tong, Shaoqing Li, Yingguo Zhu

**Affiliations:** 1 Key Laboratory of MOE for Plant Developmental Biology, College of Life Science, Wuhan University, 430072, Wuhan, PR China; 2 Key Laboratory of Molecular Biology and Gene Engineering, College of life Science, Nanchang University, Nanchang, 330047, PR China

## Abstract

**Background:**

Cytoplasmic male sterility (CMS) has often been associated with abnormal mitochondrial open reading frames. The mitochondrial gene *orfH79 *is a candidate gene for causing the CMS trait in CMS-Honglian (CMS-HL) rice. However, whether the *orfH79 *expression can actually induce CMS in rice remains unclear.

**Results:**

Western blot analysis revealed that the ORFH79 protein is mainly present in mitochondria of CMS-HL rice and is absent in the fertile line. To investigate the function of ORFH79 protein in mitochondria, this gene was fused to a mitochondrial transit peptide sequence and used to transform wild type rice, where its expression induced the gametophytic male sterile phenotype. In addition, excessive accumulation of reactive oxygen species (ROS) in the microspore, a reduced ATP/ADP ratio, decreased mitochondrial membrane potential and a lower respiration rate in the transgenic plants were found to be similar to those in CMS-HL rice. Moreover, retarded growth of primary and lateral roots accompanied by abnormal accumulation of ROS in the root tip was observed in both transgenic rice and CMS-HL rice (YTA).

**Conclusion:**

These results suggest that the expression of *orfH79 *in mitochondria impairs mitochondrial function, which affects the development of both male gametophytes and the roots of CMS-HL rice.

## Background

Maternally inherited cytoplasmic male sterility (CMS) has been described in over 150 plant species [1,2]. In most cases, the failure of pollen development in a CMS background is suggested to be associated with chimeric open reading frames (ORFs) that have arisen from unusual recombination events in mitochondria [3]. The CMS-Honglian (CMS-HL) line of rice (*Oryza sativa*) was developed by the repeated backcrossing of a red-awned wild rice (*O. rufipogon*) from Hainan Island of China with an early-maturing *indica *variety called Lian-Tang-Zao. The line is distinct both genetically and cytologically from CMS-Boro (CMS-BT) rice and CMS-Wild abortive (CMS-WA) rice [4]. Hybrid rice varieties based on CMS-HL have been widely grown in China since the beginning of this century because it performs better agronomically and produces better quality grain than those varieties based on other CMS systems. In CMS-HL rice, a chimeric mitochondrial gene called *orfH79*, located downstream of *atp6*, has been proposed as the candidate gene causing the CMS trait of Honglian rice [5]. However, no direct evidence shows that the chimeric *orfH79 *is responsible for the CMS in Honglian rice.

The mitochondrial genome carries important genetic information for a number of eukaryotic functions, including energy metabolism, development, programmed cell death and responses to oxidative stress [6]. In plants, the mitochondrial metabolism is usually disturbed by high levels of reactive oxygen species (ROS), generated in response to a range of biotic and abiotic stressors [7-9]. ROS have already emerged as an important regulator of plant development, and increasing evidence indicates that they also play a role in cell growth, since their spatial distribution affects the morphological development of plants [10]. We have previously shown that an excessive level of ROS was found during microsporogenesis in CMS-HL rice. Furthermore, the depletion of ATP and NADH and the degradation of mitochondrial genomic DNA were observed in CMS-HL rice [11,12]. However, the initial trigger of the oxidative stress response remains to be determined.

In this paper, we investigated the localization of the *OrfH79 *gene product and found that the protein is mainly present in the mitochondria of CMS-HL rice. Thus, the *orfH79 *gene was fused to a mitochondrial targeting sequence and used to transformed a Honglian maintainer line. The result showed that import of this protein into mitochondria induced cytoplasmic male sterility and reduced root growth. In addition, the expression of the transgene was also associated with excessive accumulation of ROS, a declined cellular ATP/ADP ratio, a reduced mitochondria membrane potential and a lower oxygen consumption rate. Interestingly, similar phenotypes were also observed in the YtA line. These results indicate that *orfH79 *impairs mitochondrial function, which appears to disturb the development of both male gametophytes and the roots of CMS-HL rice.

## Results

### The *orfH79 *product is present in the mitochondria of the YtA line

**Figure 1:**
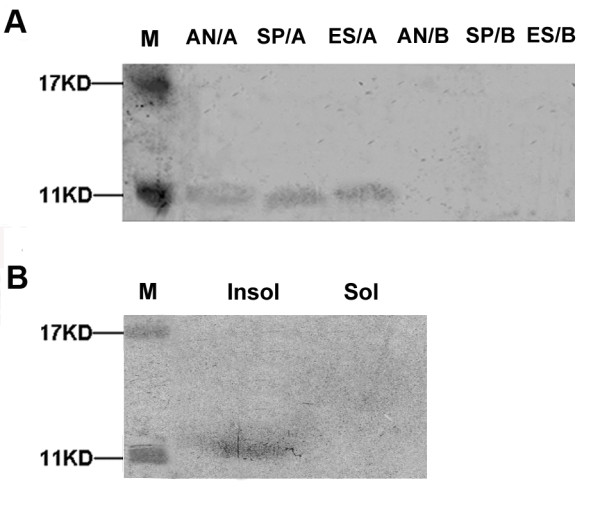
**Western blot analysis and submitochondrial location studies of ORFH79**. **A **Western blot analysis of the anther total protein extraction (AN/A,AN/B), and the purified mitochondria protein from etiolated shoots (ES/A,ES/B), and spikelets (SP/A,SP/B). Signals were obtained in the protein fraction from anthers of YtA, purified mitochondria from etiolate seedlings and spikelet of YtA. No signal appeared in any YtB tissues. ES: etiolated shoots, SP: spikelet, AN: anthers. A: YtA, B; YtB. The blot shown is representative of several independent experiments. **B **Analysis of the submitochondrial location of ORFH79. Protein samples were prepared from the purified mitochondria of YtA. Signal was observed in the insoluble protein fractions. Insol: insoluble fractions of mitochondria, Sol: soluble fractions of mitochondria.

Western blot profiles of total proteins extracted from the anthers of YtA, as well as mitochondrial proteins isolated from the etiolated seedlings and spikelets were distinguished by the presence of an ~11 KD protein. This product was detectable among the total anther protein sample and the mitochondrial protein samples from the etiolated seedling and spikelet, but not among the total protein samples form either the etiolated seedling and spikelet (Additional file 1), or in any samples extracted from YtB (Fig. 1A). To rule out the possibility that the signal in the YtA samples was non-specific, western blot were performed using only the secondary antibody (without the primary antibody). In these negative controls, the ~11 KD band not appear (data not shown). Meanwhile, electron microscope immunocytological analysis also showed ORFH79 protein were mainly localized in mitochondrial of YtA but not in YtB (Additional file 2).Thus, ORFH79 appears to strongly enriched in the mitochondria of YtA, and at a particularly high level in the anther. The *orfH79 *gene sequence predicts a protein with a molecular mass of 9 KD, instead of the 11 KD band that we observed. This discrepancy may have been due to anomalous gel migration. The submitochondrial distribution of ORFH79 was also investigated. As shown in Fig. 1B, ORFH79 protein appeared to be mainly associated with the insoluble protein fractions (Fig. 1B). Taken together, these results indicated that ORFH79 protein is mainly present in the mitochondria of YtA both in anthers as well as in vegetative tissues, and attached to the mitochondrial membranes.

### Expression patterns of ORFH79 in anther and root tip of YtA line

**Figure 2:**
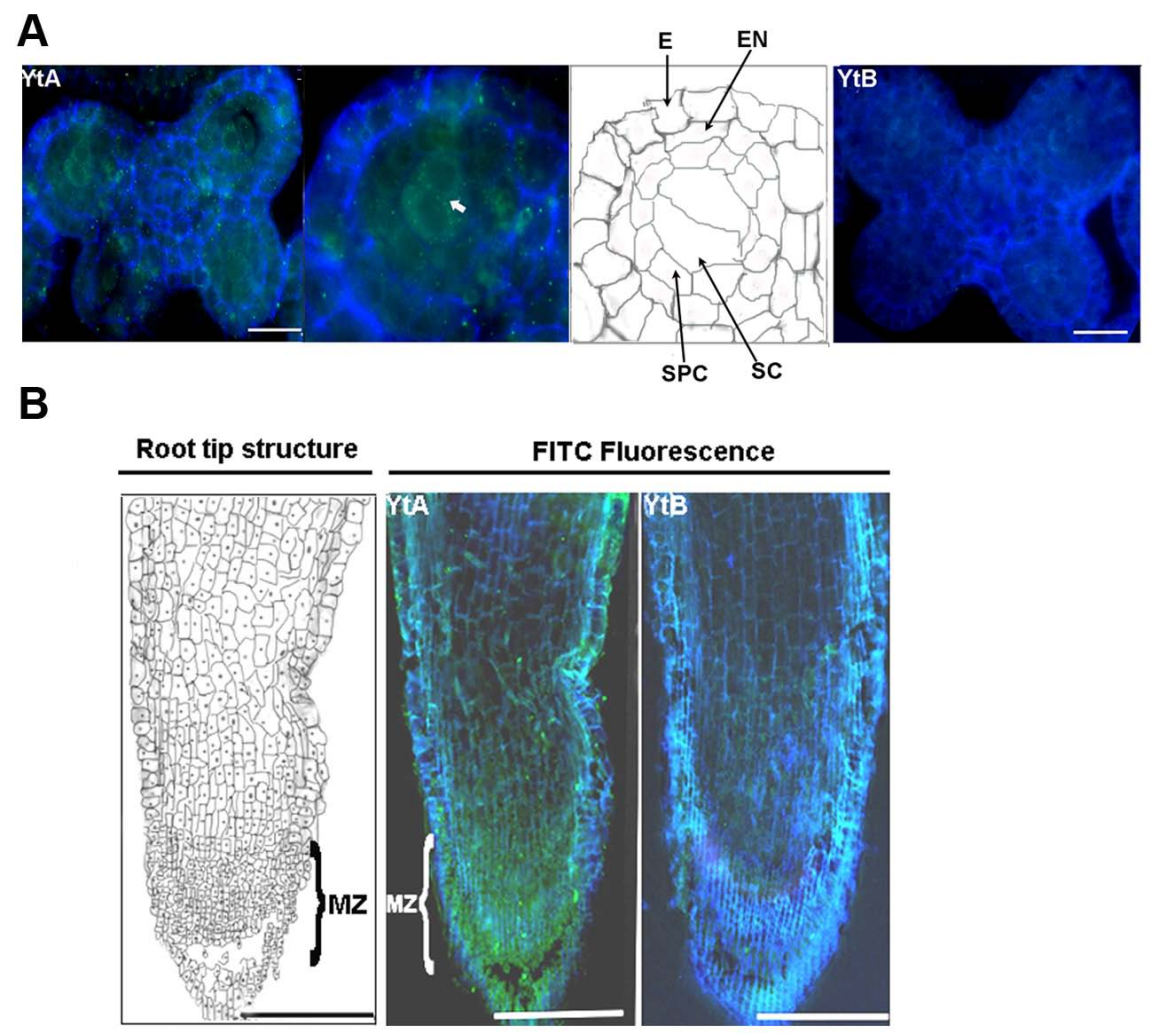
**Expression pattern of ORFH79 in anther and root tip of YtA**. Positive signals are green (FITC), while autofluorescence is pseudo-colored blue. **A **Expression pattern of ORFH79 in a premeiotic anther. The signal has accumulated in the sporogenous cells (white arrow) of the premeiotic YtA anther. In the premeiotic YtB anther, no ORFH79 signal was present (bar represents 20 μm). **B **Expression pattern of ORFH79 in root tip. In the 5-day-old YtA root tip, signals were found to mainly accumulate in the meristematic region (MZ); In the five day old YtB root tip, the signal was barely detectable (bar represents 100 μm). E, epidermis; En, endothecium; SPC: Secondary parietal cell; Sc, sporogenous cell; MZ, meristematic region.

To analyze the expression pattern of ORFH79 in the anther and root tip, immunofluorescence detection was used. In sections of the premeiotic anthers of YtA, ORFH79 was concentrated in the sporogenous cells, and was present at a gradually declining level in the secondary parietal cells, endotheciumnd and epidermis. In control sections of YtB anther, only the background signal was detected (Fig. 2A). In the sections of root tip, a strong signal was detected in the meristematic zone of the YtA line, while a weak signal was observed in other regions of the YtA root tip. In contrast, only a low background signal was observed in YtB line (Fig. 2B). These results indicate that the distribution of ORFH79 protein is not uniform in anther and roots in the YtA line.

### A chimeric *orfH79 *transgene induces CMS

**Figure 3:**
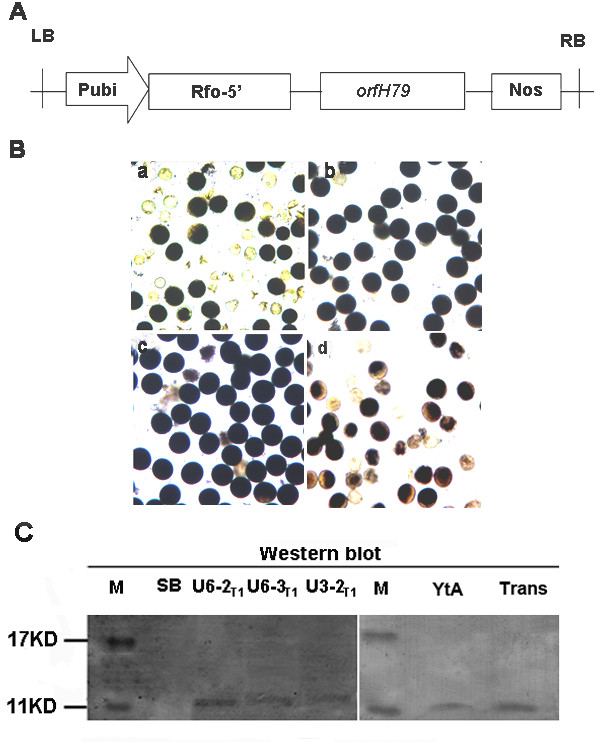
**The *orfH79 *transgene cassette, and its effect *in planta *on pollen abortion**. **A **The structure of the binary vectors containing *orfH79. *Pubi: the maize ubiquitin promoter, *Rfo*-5': a mitochondrial transit signal from the *Raphanus sativus *restorer-of-fertility (*Ppr-B*) gene. **B **The effect of the transgene on pollen morphology. Darkly stained and lightly stained grains represent, respectively, fertile and sterile pollen. **a **Pollen from a T0 transformant carrying *orfH79*. **b **Pollen from ShijinB. **c **Pollen from a T0 transformant carrying an empty plasmid. **d **Pollen from a T1 transformant carrying an inserted *orfH79 *gene. **C **Western blot analysis of the mitochondria-enriched protein of the callus. SB, mitochondria protein from callus of ShijinB. U6-1_T1_,U6-2_T1 _and U3-2_T1_, mitochondria protein of callus derived from the three T1 progeny transgenic seeds, respectively. YtA, purified mitochondria protein from etiolated shoots of YtA line. Trans, mitochondrial protein of callus from T1 progeny transgenic seeds.

To provide a direct genetic evidence that *orfH79 *can cause the male sterility of CMS-HL, a cheimeric ORFH79/mitochondria transit peptide was used for a transgenic experiment. Expression cassettes pCON containing a constitutive promoter (Pubi), a mitochondrial transit peptide sequence and *orfH79 *was transferred into ShijinB (SB), a japonica maintainer line of CMS-HL rice (Fig. 3A). Nineteen independent events were obtained and 15 transgenic events were confirmed by PCR firstly (data not shown). Furthermore, the 15 transgenic events appeared to display the typical phenotype of gametophytic male sterility, showing an approximately 1:1 ratio of fertile (normal) vs. sterile (abortive) pollen grains (Fig. 3a). The seed-sets ranged from 50% to 70% in those T0 transgenic plants, indicating that the pistil was unaffected (data not shown). To further confirm that *orfH79 *causes the gametophytic male sterility of Honglian rice, three independent T0 transformants (U6-2, U6-3 and U3-2) were randomly chosen in order to analyze the segregation of the transgene in the T1 progeny. The segregation of the transgene among the T1 progeny (Table 1) fit a 1:1 ratio, and the pollen of T1 progeny carrying *orfH79 *also showed a semi-male-sterile phenotype (50% abortion pollen) similar to that of T0 progeny (Fig. 3d). If the presence of *orfH79 *was not correlated with pollen sterility, the transgene segregation in T1 progeny would be 3:1 rather than the 1:1 ratio that we observed. Thus, these results indicate that the expression of ORFH79 induced the abortion of pollen and that the transgene was transmitted through the female germ.

**Table 1:** Segregation of the T1 transgenic plant with *orfH79*

Plant lines	Number of positive plant	Number of negative plants	χ^2^value (for 1:1 segregation)	P
U6-2	15	13	0.14	0.70-0.90
U6-3	10	10	0.05	0.90-0.95
U3-2	17	13	0.3	0.50-0.70

The presence of transgene was determined using PCR. Positive plants: T1 progeny with *orfH79 *insert, showing a semi-male-sterile phenotype (50% abortion pollens); Negative plants: T1 progeny without *orfH79 *insert, and showing a fertile phenotype.

### Molecular evidence of the expression of ORFH79 in the transgenic plants

To provide further molecular evidence that ORFH79 can cause the male sterility phenotype in the transgenic lines, expression of the transgene at the protein level was assayed by western blot. The mitochondria-enriched protein fraction was extracted from the callus that had been derived from the transgenic lines (U6-2, U6-3, U3-2) and probed with anti-ORFH79 antibody. An 11 KD band similar to that observed in YtA was detected in the transgenic callus, but not in the shijing B callus (Fig. 3C). This confirmed that the transit peptide fused to ORFH79 was properly processed and that ORFH79 was imported into mitochondria in these transgenic lines. The results provided direct molecular evidence demonstrating that the presence of ORFH79 in mitochondria corresponds to the male sterile phenotype of CMS-HL rice.

### Mitochondria activity assay in YtA line and transgenic plants expressing ORFH79

**Figure 4:**
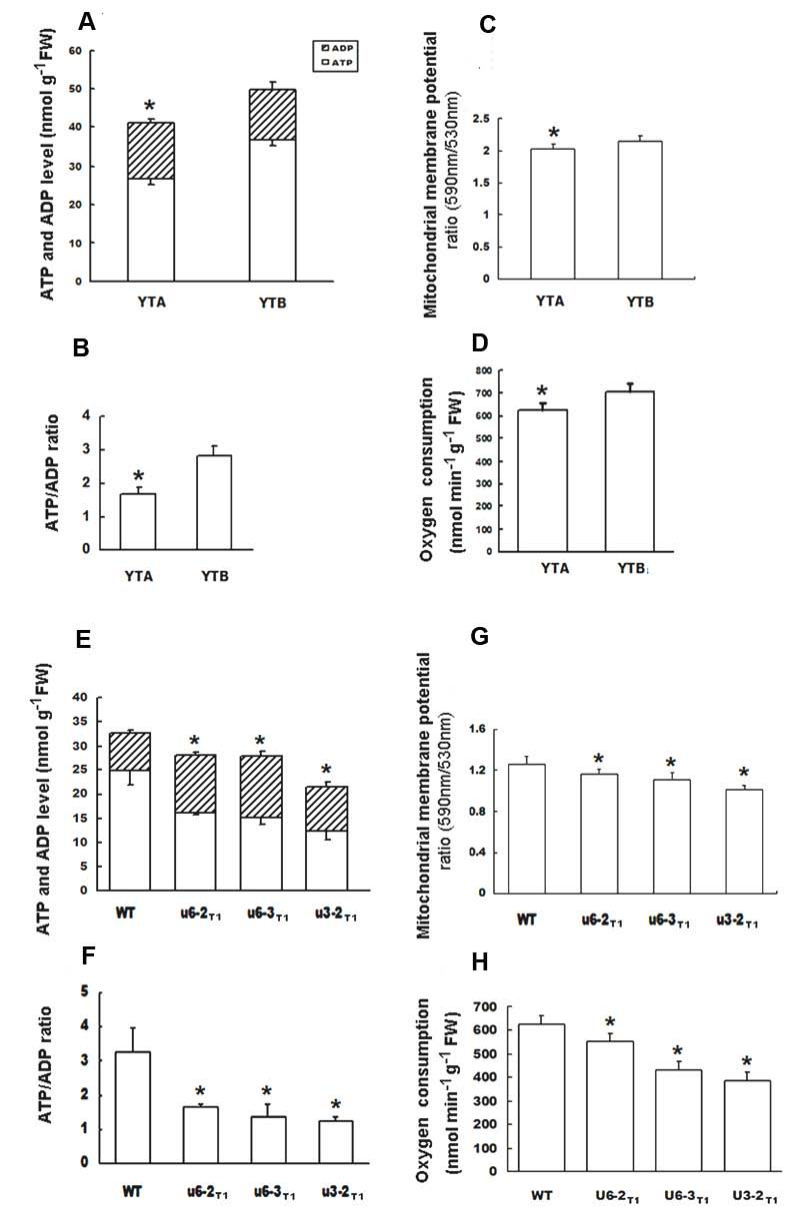
**ATP and ADP measurements, mitochondrial membrane potential analysis and respiration assay in YtA line and transgnic plants**. **A **ATP and ADP levels from the root of YtA and YtB. **B **ATP/ADP ratios of the plant materials. **C **Purified mitochondria were isolated from the root of YtA and YtB, and the mitochondrial potentials were assayed. **D **Respiration rates in roots from YtA and YtB. Experiments were replicated three times. E ATP and ADP levels from 3-day-old etiolated seedlings of transgenic and non-transgenic rice. **F **ATP/ADP ratios of the transgenic plants and non-transgenic plants. **G **Mitochondrial membrane potential of these plant materials. **H **Respiration rates in roots from wild-type and transgenic plants at one week. Three individual samples of each line were measured. The data are given as mean ± SE (*n *= 3). *, a to d indicate the difference between YtA and YtB is significant (p < 0.05). *, e to h indicate the difference between transgenic and wild type plants is significant (p < 0.05). U6-1_T1_,U6-2_T1 _and U3-2_T1_, three independent T1 progeny transgenic plants.

Since ORFH79 is present mainly in mitochondria of YtA, and importing ORFH79 into mitochondria can induce the CMS trait, which inspired us to focus on the mitochondrial activity. Thus, we measured ATP and ADP content, the mitochondrial membrane potential and the respiration rate both in the YtA line and transgenic plants. It was found the ATP content in the male-fertile line (YtB) was higher than that in the male-sterile line (YtA) (Fig. 4A), and meanwhile, the ATP/ADP ratio in YtB line was about 2-fold higher than that in YtA line (Fig. 4B). The mean of mitochondrial membrane potential in YtA line was found to be 5.7% lower than that of YtB (Fig. 4C), and the oxygen consumption rate in YtA roots was decreased by 11.8% compared with that in YtB roots (Fig. 4D). Three independent transgenic plants (U6-2, U6-3 and U3-2) that had been determined to be male sterile and confirmed to contain ORFH79 protein were chosen to do the parallel assay. The result showed that the mitochondrial membrane potential of the transgenic plants was reduced by 7.1-12.1% (Fig. 4G), while the ATP+ADP contents was reduced by 20.1-36.3% (Fig. 4E) and the average of ATP/ADP ratio was decreased by 59.5% in contrast to the wild type (Fig. 4F). In addition, the respiration rates in transgenic plant roots were found to be significantly decreased by 11.2-38.3% compared with the wild type.

**Figure 5:**
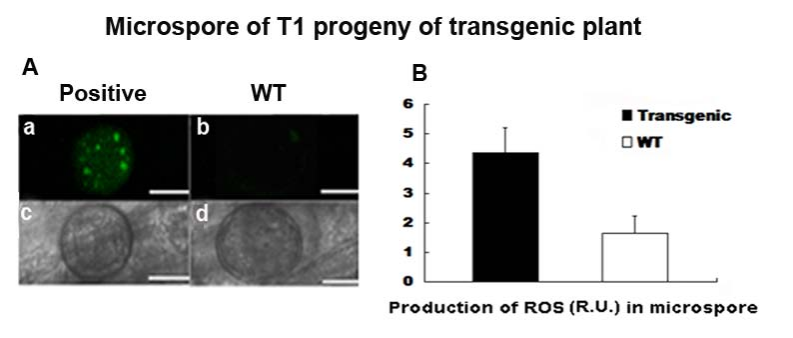
**Analysis of intracellular production of reactive oxygen species (ROS) in transgenic microspores**. **A **Anthers were incubated in 10 μM H_2_DCFDA for 10 min, and an image of ROS production was captured using confocal microscopy. **a **and **c **, Confocal images of T1 transformants carrying *orfH79*. **b **and **d **, Confocal images of non-transgenic plants. **a **and **b **, Fluorescent field. **c **and **d **, Bright field (bar represents 500 μm). **B **Production of ROS in microspores of transgenic and wild type plants. ROS content in microspores was quantified after compiling the projections from eight confocal sections of 0.9 μm each (bar represent 100 μm). R.U, relative unit. The data are given as mean ± SE (n = 3).

Based on our previous investigation, excessive ROS content was found during microsporogenesis in CMS-HL rice [11]. To detect whether the transgenic plants also present excessive ROS levels during microsporogenesis, we measured the ROS content in the microspore of the transgenic plants using 2',7'-dichlorodihydrofluores-cein diacetate (H_2_DCFDA), a dye that is sensitive to ROS. Strong fluorescent intensity was observed in microspores of the transgenic plants, whereas much weaker fluorescence intensity was detected in wild type (Fig. 5A-a, b and 5B).

Taken together, these physiological features in the transgenic lines mimic those in the YtA line, this provided another evidence that *orfH79 *is responsible for the CMS trait of YtA. Furthermore, these data showed that the mitochondrial activity both in YtA line and transgenic plants are down-regulated, suggesting that accumulation of ORFH79 in mitochondria impairs their normal function.

### The transgenic expression of *orfH79 *retards root growth

**Figure 6:**
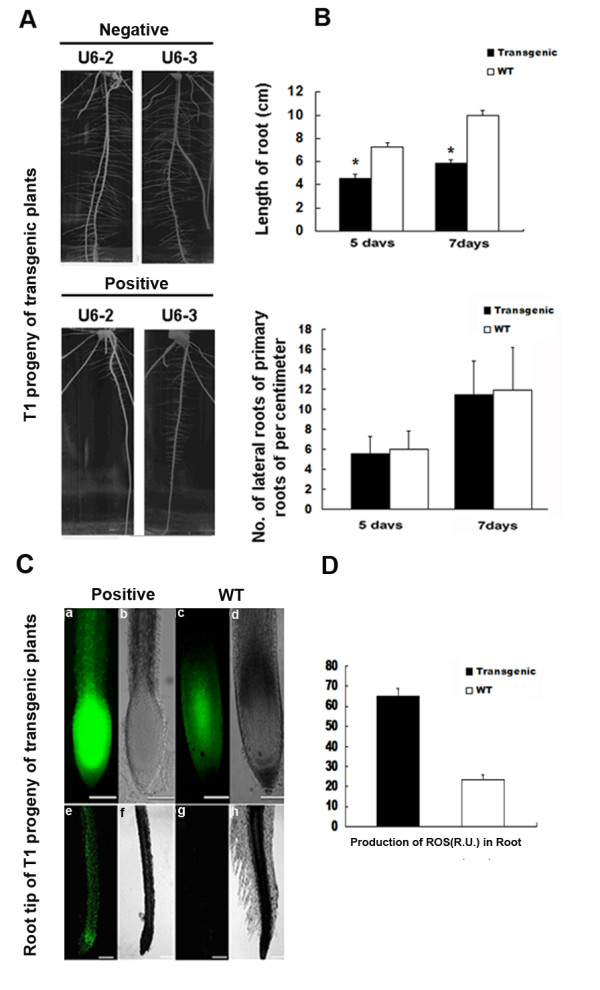
**Root morphology and intracellular production of reactive oxygen species (ROS) analysis in root tip of transgenic rice lines carrying *orfH79***. **A **Two independent T1 progeny transgenic seedlings (U6-2, U6-3) which are eight days after germination on 1/2 MS in vertical plates. **B **Length of the primary roots of 5-day- old and 7-day-old seedlings, and the number of lateral roots of five and seven day old seedlings were recorded and analyzed. The data represent the mean value ± SE from 8 (positive and negative) plants. *, indicates that difference between transgenic and wild type plants is significant (p < 0.05). Positive: transgenic plant carrying *orfH79*; negative: non-transgenic plant. **C **Seedlings were grown on 1/2 MS in vertical plates for five days. Root tips were incubated in 10 μM H_2_DCFDA for 10 min, and an image of ROS production was captured using confocal microscopy. **a b e **and **f **, Confocal images of T1 transformants carrying *orfH79*. **c d g **and **h **, Confocal images of non-transgenic plants. **a **, **c **, **e **, **g **, Fluorescent field. **b **, **d **, **f **, **h **, Bright field (bar represents 500 μm). **D **Production of ROS in roots of transgenic and wild type. plants. ROS content in root was quantified after compiling the projections from 32 confocal sections of 0.75 μm each. R.U, relative unit. The data are given as mean ± SE (n = 3).

In this study, we found that root growth of the transgenic plants were slower than that of the wild type. Thus, root growth and development of the T1 transgenic progeny seedlings (U6-2, U6-3) was investigated in more detail. The length of primary roots of the independent transgenic seedlings was on average 2.6 cm shorter than that of wild type seedlings growing at the same stage. Furthermore, in the transgenic plants, the appearance of lateral roots was delayed by about two days (Fig. 6A, B). The total number of lateral roots in transgenic seedlings was less than that of the wild type at both five and seven days after germination. However, the number of lateral roots per centimetre was almost equal to that of the wild type. Thus, it is likely that the initiation of lateral roots growth in transgenic plants was not defective. These findings demonstrate that constitutive expression of *orfH79 *retards root growth in transgenic plants.

### Excessive ROS levels in the roots of both transgenic plants and the YtA line contribute to retardation of root growth

Previous research has shown that ROS plays an important role in plant root development [10] and we have found excessive ROS in the microspore of the *orfH79 *transgenic plants. Thus, we analyzed the production of ROS in the root tips of transgenic plants using the oxidant-sensitive H_2_DCFDA. The intensity of H_2_DCFDA fluorescence in the transgenic plant root tip was observed to be higher than that in the wild type (Fig. 6C-a, c and 6D). Furthermore, the appearance of root hair was observed to be delayed in transgenic plants compared with wild-type plants (Fig. 6e, f, g, h).

**Figure 7:**
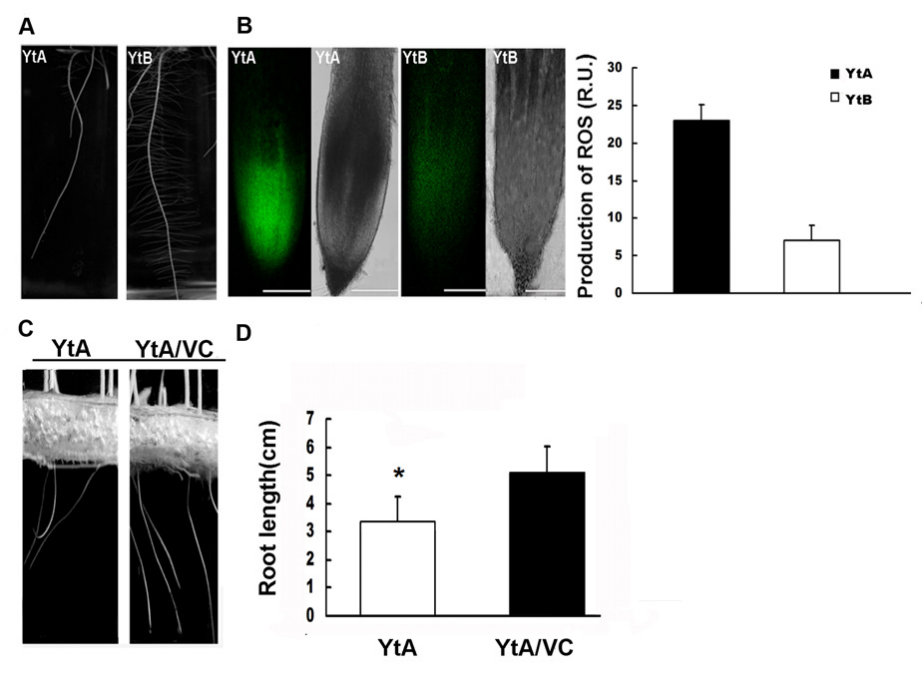
**Root morphology and ROS level in YtA and YtB**. **A **3-day-old root of YtA and YtB on 1/2 MS vertical plates. **B **Confocal micrographs illustrating ROS production in roots of YtA and YtB (bar represents 100 μm). Production of ROS was quantified after compiling the projections from 23 confocal sections of 0.74 μm each. R.U, relative unit. The data are given as mean ± SE(n = 3). **C **The effect of exogenous supply of ROS scavengers. 5-day-old YtA seedling root and 5-day-old YtA seedling root treated with 100 nM VitaminC. **D **Length of the primary roots of 3-day-old seedlings assay in the absence or in the presence of VitaminC. The data are given as mean ± SE (n = 3).

Similarly, we analyzed the level of ROS in root tips of YtA and YtB. Higher fluorescence intensity was detected in the root tip of YtA than in YtB (Fig. 7B). Furthermore, the primary root and lateral root growth were observed to be slower in YtA compared with YtB (Fig. 7A).

To address the question as to whether the root growth inhibition observed in YtA was brought about by a higher level of oxidative stress, the effect of adding the ROS scavenger Vitamin C to the culture solution was explored. Interestingly, the inhibition of YtA root growth was observed to be rescued when 100 nM Vitamin C was supplemented in the solution (Fig. 7C, D). Taken together, these results imply that root growth retardation in the YtA line is associated with the excessive ROS content.

## Discussion

### *orfH79 *is responsible for the gametophytic male sterility of CMS-HL

CMS is a widespread phenomenon in plants, and is often associated with abnormal mitochondrial ORFs. Although *orfH79 *was proposed as a candidate gene corresponding to the male sterility of CMS-HL rice, direct evidence for this was lacking. Our experiments showed that the transgenic expression of *OrfH79 *induces the abortion of about 50% of the pollens, indicating only the pollens carrying expressed *orfH79 *were disrupted, and pollen abortion occurs after meiosis. This is a typical feature of gametophytic male sterility in plants and this feature is associated with the segregation of the transgene in the T1 progeny. Furthermore, the physiological features of the transgenic lines expressing *orfH79*, including reduced ATP/ADP ratio, high ROS content, decreased mitochondrial membrane potential and lower respiration rate mimic all features of the HL-CMS line (YtA). Therefore, we believe that *orfH79 *is responsible for the gametophytic male sterility of CMS-HL rice.

In CMS Boro rice, *orf79*, which is probably allelic to *orfH79*, has been identified as the gene responsible for male sterility [13]. *orfH79 *and *orf79 *share 98% identity with one another at the nucleotide level. Only 5 nt differ, but each of these differences results in a distinctly different codon. However, both the appearance of the aborted pollen and the timing of pollen abortion differ markedly between these two CMS types. In CMS-BT rice, pollen abortion occurs at the tri-nucleate stage and is stainable with 1% I_2_-KI solution, revealing spherical, black pollen grains. While in CMS-HL rice, pollen aborts at the di-nucleate stage and is not stainable with 1% I_2_-KI solution, revealing spherical, blank pollen grains (Additional file 3). The mechanisms by such similar genes can cause such divergent phenotypes remain to be determined.

Similarly to the CMS associated proteins such as URF13 in CMS-T maize [14] and ORF129 in CMS wild beet [15], ORFH79 was observed to accumulate mainly in the mitochondria in both vegetative and reproductive tissues. At the same time, the accumulation of high levels of ROS, a significantly decreased adenylate content as well as ATP/ADP ratio, reduced mitochondrial membrane potential and a lower oxygen consumption rate were found both in the YtA line and in the transgenic plant in which ORFH79 was imported into mitochondria. Thus, the results provided here demonstrated that accumulation of ORFH79 in mitochondria impairs the mitochondrial function. In fact, dysfunctional mitochondria distribute throughout the plant but only the pollen function is lost, suggesting that the mitochondrial activity is strictly required for gametophyte development during microsporogenesis. This idea is in agreement with the hypothesis that the abnormal mitochondria in the CMS line cannot meet the increasing demand for energy during microsporogenesis [15,16]. However, how the aberrant ORFH79 protein affects mitochondrial function remains a subject for further investigation.

### Retarded root growth is associated with excessive ROS caused by expression of *orfH79*

In previous studies, significant attentions have been focused on reproductive development in the study of CMS. Detailed studies on vegetative development have not been performed, especially on roots growing underground. However, in the CMS lines of *Brassica napus *and *Nicotiana tabacum*, all tissues except for green leaves showed lower ATP levels than their respective maintainer lines. This reduced energy availability had limiting effects on plant growth [17,18]. Nevertheless, reduced plant height and retarded vegetative growth were also reported in other CMS systems such as *B. juncea *[19], *N. tabacum *cybrids [20], and *T. aestivum *[21].Although some differences in alloplasmic lines have been observed, retarded root growth and reports of chimeric ORF expression associated with the retardation of vegetative growth in CMS plants have not been described until now. In our transgenic experiments, transgenic plants with *orfH79 *under the control of a constitutive promoter (Pubi) showed retarded root growth similar to the YtA line. However, the root development of the transgenic plants with *orfH79 *under the control of a pollen specific promoter was indistinguishable from that of the wild type, despite the fact that the transgenic plants also revealed an aborted pollen phenotype (data not shown). Therefore the products of such a CMS-associated ORF may disturb not only to the gametophyte development but also root growth.

ROS play important physiological roles in plants, especially in signalling cell division and expansion, root growth and root hair development [22,23]. However, excessive levels of ROS produce oxidative stress and inhibit plant root growth, as shown in the case for the inhibition of *Arabidopsis thaliana *root tip elongation, which was inhibited by exogenously applied hydrogen peroxide [24]. In the pea root, high levels of exogenous aluminum ions trigger excessive amounts of ROS production, leading to respiration inhibition, ATP depletion and the inhibition of root elongation [25]. A similar effect has also been reported in rice roots: lead toxicity stunted rice root growth via abnormal ROS production [26]. In our studies, an excessive amount of ROS production was found both in the roots of YtA line and in transgenic plants. Moreover, the slow elongation of the primary root and lateral roots of YtA can be rescued by a known scavenge of excessive ROS, Vitamin C. Taken together, these results suggested that the excessive ROS content caused by the expression of *orfH79 *is responsible for the retarded root growth.

**Figure 8:**
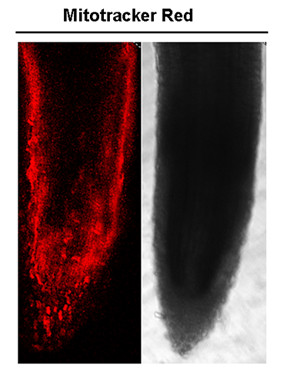
**Confocal projections of YtA root tip after incubation in Mitotracker Red**. Seedlings were grown on half-strength MS vertical plates for five days, then the root tips were incubated in 10 μM Mitotracker for 40 min, and imaged by confocal microscopy. The red fluorescence is more concentrated in the meristematic region of the root tip.

As revealed by immunofluorescence, ORFH79 preferentially accumulates in the sporogenous cells and root tip meristematic zone in the YtA line. Lee and Warmke have previously reported that a 40- and 20-fold increases in mitochondrial number respectively in maize tapetal and sporogenous cells during microsporogenesis [27]. Furthermore, we also observed that the density of mitochondria is higher in the root tip meristematic zone than that in other regions of the root (Fig.8). Thus, the distribution pattern of ORFH79 in anther and root is in accordance with the density distribution of mitochondria in these tissues. From this observation, we infer that the large number of mitochondria accumulated in the meristematic zone of these young tissues may amplify the consequences of mitochondrial dysfunction caused by *orfH79*. This may be a critical reason responsible for the inhibition of development of these tissues.

### Conclusion

In summary, we found ORFH79 accumulated mainly in mitochondrial and imported the protein into mitochondria induced male sterile and retarding root growth. In addition, the expression of the transgene was also associated with declined cellular ATP levels, reduced mitochondria membrane potential, and excessive accumulated ROS content. Furthermore, similar phenotypes were also observed in YtA line. These results indicate that *orfH79 *impair the mitochondria function, which may disturb the development of both male gametophyte and root of CMS-HL rice.

## Methods

### Plant materials

A CMS-HL line, YuetaiA (YtA), and its maintainer line of YuetaiB (YtB, Indica) and ShijinB (SB, japonica) were used in this study. T0 progeny transgenic plants were grown at 25°C under 16 h of daylight in a greenhouse. For physiological analysis, plants were grown under a controlled temperature of 23°C and at 70% relative humidity.

### Plasmid construction and rice transformation

The sequence of the gene coding for the mitochondrial transit peptide was derived from RFo (Genbank accession EF472240.1) [28]. To enhance the expression of *orfH79 *in the nuclear genome of rice, the fusion between this transit peptide sequence and *orfH79 *was synthesized using rice-preferred genetic codons based on the Codon Usage Database that provided by Kazusa DNA Research Institute http://www.kazusa.or.jp/codon/. The fusion was synthesized by overlap extension PCR [29]. Four long primers with 10 bp of overlap were synthesized and PCR reactions were carried out as described by [30]: s1, 5'-atgaccaacctgctccgctggctcttctccaccacccgcggcaccaacggtctcccatactt catcttcg-3';s2, 3'aagtagaagccacagc accacccgcc gcgggacaaaagcgaaacgatc atagtcggggagacatg-5'; S3, 5'-cctctgtacgaccccgctctactggacaagatcatagat cataacataaaggccggctaccctatagagg-3'; S4, 3'-gatatctccacctgaaccccgtga gataagcacaccagaagggattcac T-5'. The synthetic gene was ligated to the OsPMP41 [31] to form an intermediated construct designated pGUS1. This was then digested with *Hin*dIII and *Bam*HI and ligated to a strong constitutive promoter Pubi (the maize ubiquitin promoter). The entire expression cassette (Pubi:: synthetic gene:: Nos) was digested with *Hin*dIII and *Eco*RI and ligated into a binary vector pCAMBIA1301, that had been digested with the same enzymes. The resulting constructs were designated pCON. The *japonica *rice variety ShijinB was transformed using *Agrobacterium tumefaciens EHA-105*, following the published procedures by Hiei et al. [32].

### Preparation of proteins

Total protein was extracted from etiolated shoots, spikelets and anthers of YtA and YtB. Tissues were ground into a fine powder in liquid nitrogen. 400-500 mg of tissue were extracted in 1 ml of extraction buffer (66 mM Tris-Cl, pH 6.8, 2% SDS, 2% v/v 2-mercaptoethanol) at room temperature followed by centrifugation for 20 min at 40000 × g to remove the insoluble fraction. Proteins were precipitated at -20°C for 1 h by the addition of 1 ml of an 8:1 mixture of ice-cold acetone and trichloroacetic acid with 0.1% v/v 2-mercaptoethanol. The supernatant was discarded after centrifugation at 18000×g for 15 min at 4°C, and the pellet was washed in 1 ml of ice-cold acetone. The remaining acetone was removed by air drying at room temperature. The resulting pellet was redissolved in the protein extraction solution. Purified mitochondrial proteins were extracted from the same tissues and transgenic callus mitochondria as described by Wan et al. [11]. The YtA line mitochondria protein were separated into soluble and insoluble fractions according to a method described by Uyttewaal et al. [33]

### Antibodies and western blotting analysis

A polyclonal antibody against ORFH79 was obtained from a commercial service (Alpha Diagnostic International, San Antonio, Tex., USA) by injection of a synthetic peptide, comprising residues 37 to 55 of ORFH79 into rabbits. The specificity of the antiserum was tested by western blot the ORFH79 protein expressed in *E. coli *(Additional file 4). Equal amounts of protein from each fraction were separated by 18% SDS-PAGE gel at 4°C, and then transferred onto an Immobilon-PSQ transfer membrane (PVDF type; Millipore) at 80 V for 40 min. The membrane was incubated in 5% w/v non-fat milk, 0.05% v/v Tween-20, in phosphate buffered saline (PBS) for 1 h, washed for 10 min three times in PBST (PBS, 0.05% Tween-20), and incubated in a 1:500 dilution of rabbit antiserum overnight at 4°C. After four washes with PBST, the membrane was incubated with goat anti-rabbit IgG conjugated with alkaline phosphatase (AP) in PBST solution for 2 h. After four 10-min washes in PBST, signal was detected according to a method described by Yue et al [34]. Briefly, the membrane was washed with AP 7.5 buffer (0.1 M Tris-HCl pH 7.5, 0.1 M NaCl, 2 mM MgCl_2_) twice, and once with AP 9.5 buffer (0.1 M Tris-HCl pH 9.5, 0.1 M NaCl, 50 mM MgCl_2_) for 10 min each. Then the membrane was incubated with 2.5 mg nitroblue tetrazolium (NBT; Promega) and 1.25 mg 5-bromo-4-chloro-3-indolyl phosphate (BCIP; Promega) in 7.5 ml of AP 9.5 buffer at room temperature until the signal appeared. Finally, TE buffer (10 mM Tris-HCl pH 8.0, 1 mM EDTA pH 8.0) was added to stop the reaction. As a negative control, a western blot was simultaneously processed in which only the secondary antibody was used.

### Immunofluorescence analysis of the *orfH79 *product in anther and root tip

Five-day-old root tips and premeiotic anthers of YtA were dissected, fixed and sectioned, as described by Paciorek et al. [35]. After dewaxing and rehydration, the sections were washed six times in PBST, 5 min each wash. They were then blocked in 0.5% (w/v) BSA and 5% (v/v) normal goat serum in PBST for 30 min at room temperature, and finally incubated overnight at 4°C in a 1:100 dilution of the primary antibody in PBS, 5% (w/v) non-fat milk, 0.05% (v/v) Tween-20. After six washes of 5 min each in PBST, the slides were incubated with goat anti-rabbit FITC-conjugated secondary antibody (1:50) at room temperature for 2 h, and subjected to five 10-min washes in PBST. Hybridization signals were visualized and captured by a cool CCD camera mounted on an Olympus BX51 microscope.

### Root length and the number of lateral root assay

T1 seeds of the transgenic and non-transgenic plants were sown on 1/2 MS medium containing 0.3% (w/v) phytagel (Sigma, St Louis, MO, USA). Images of 5-day-old and 7-day-old roots were collected, and length and lateral root numbers were recorded. Eight to ten roots were measured in each of three to five replicates.

### Detection of ROS

T1 seeds of three independent transgenic and non-transgenic plants and seeds of YtA and YtB were de-husked, sterilized and then germinated on 1/2 MS medium containing 1.0% (w/v) phytagel gel. Five-day-old seedling root tips were harvested. ROS detection was performed as previously described by Umbach et al. [36]with slight modifications. Briefly, root tips were excised and placed in 4 ml of 1 mM Tris-Cl, pH 8.0, and gently shaked at 23°C in dark for 30 min. Then the roots were incubated with 10 μM 2',7'-dichlorodihydrofluorescein diacetate (H_2_DCFDA[Sigma]) in 1 mM Tris-Cl buffer (pH 8.0) for 10 min. After a briefly rinsing with the Tris-Cl buffer to remove the dye, the roots were imaged with a confocal microscope. Optical filters were set to the maximum absorption wavelength of 488 nm and the emission wavelength of 530 nm. The experiment was performed in triplicate. For anther ROS detection, the anthers were punctured by a dissecting needle so that the H_2_DCFDA could penetrate easily. Image analysis and quantification of the fluorescence intensity were performed by the Leica LCS Lite software.

### ATP and ADP measurements

0.5 g fresh weight (FW) of one week old root tip from YtA and YtB were collected respectively. Three-week old T1 progeny transgenic seedlings, as well as the negative plants that had been segregated from T1 progeny as a negative control were etiolated for three days. Root tips and etiolated plants (0.5 g FW) were ground in liquid nitrogen. ATP and ADP were extracted using 4.5% HCLO_4 _according to Botrel and Kaiser [37] and measured by the luciferin-luciferase method [38,39] following the protocol of the ATP detection kit (Beyotime, China). ADP was converted to ATP by pyruvate kinase (EC2.7.1.40) (Sigma). The ATP content was assayed by luminescence in a Multifunctional Microplate Reader (SpectraMax M2, MDC). Three independent transgenic lines were used in this experiment, and each measurement was repeated three times.

### Measurement of mitochondria membrane potential (ΔΨ_m_)

T1 seeds of transgenic and non-transgenic plants were de-husked, sterilized, and germinated on 1/2 MS medium at 28°C in the dark. Five-day-old etiolating seedling were harvested for protoplast isolation. About 1 g of young seedlings was sliced into 0.5 mm pieces and placed into a 10 M CPW solution containing 0.7 M mannitol for 2 h for preplasmolysis. The pieces were then incubated in digesting medium (1.5% cellulose enzyme [Sigma], 1% Hemicellulase [Sigma], 0.5% pectolyaser [Onazuk, Japan] in 10 M CPW) for 4 h at 30°C in the dark. Protoplast were purified as described by Parys et al. [40]. The integrity and activity of the protoplasts were checked by staining them with neutral red and observing them with a microscope. JC-1 was used a measure the mitochondrial potential (ΔΨ_m_) as described by Simeonova et al. [41]. The protoplasts (10^6 ^ml^-1 ^) were suspended in JC-1 buffer supplied by the JC-1 kit (Beyotime, China). Briefly, the protoplasts were treated with 10 μg ml^-1 ^of JC-1 in the dark for 20 min, washed twice with JC-1 buffer and analyzed on a Multifunctional Microplate Reader (SpectraMax M2, MDC) with excitation at 490 nm and emission at 530 and 590 nm, respectively. ΔΨ_m _was determined by the ratio of fluorescence intensity at 590 nm to 530 nm in triplicate. With the same kit, mitochondria isolated from the root of YtA and YtB were suspended in JC-1 buffer. Then, 10 μg ml^-1 ^of JC-1 was added. After incubation in the dark for 10 to 15 min, then the mitochondria membrane potential of YtA and YtB was assayed by calculating the ratio of fluorescence intensity at 590 nm to 530 nm in triplicate.

### Root respiration measurements

Roots from five-day old YtA and YtB, and one-week-old transgenic and wild type seedlings were prepared respectively. The roots were briefly rinsed in a buffer (10 mM MES-KOH pH 6.5) to remove dead cells and then immediately transferred to the temperature-controlled chamber of a chlorolab 2 electrode (Hansatech, UK) containing 2 ml of buffer. Respiration rate was measured by detection of oxygen consumption at 25°C.

### Vitamin C (VC) treatment

Seeds of YtA and YtB were de-husked, sterilized, and germinated in culture solution [42] containing 100 nM vitamin C at 28°C for five days. The length of the primary roots and the development of lateral roots were measured. Five independent plants for each sample were measured respectively.

### Assays for mitochondrial distribution in root tip

Five-day-old roots tips were harvested, then the root tips were stained with 10 μM MitoTracker red (Molecular Probes) for 40 min and then washed in water for 20 min. Image analysis was performed using the Leica LCS Lite software.

### Statistical analysis

P-values were calculated using Microsoft Excel (2003 version). All error bars in the graphs indicate standard errors of the mean. The statistical significance of the experimental data was determined using the two-sided Student's *t*-test.

## Authors' contributions

SQL and YGZ designed the study. XJP performed most of the experiments and wrote the manuscript. KW helped in carrying out western blot, CFH helped in purifying the mitochondria, YLZ helped in analyzing the data, TW helped in detecting the ROS content, JY and JPT helped in planting the transgenic rice. All authors read and approved the final manuscript.

## Supplementary Material

Additional file 1 **Western blot analysis of the total protein extracted from anthers, etiolated shoots, and spikelet without mitochondria purification in the YtA line.**  AN: anthers, ES: etiolated shoots, SP: spikelet Click here for file

Additional file 2 **Immunogold localization of ORFH79 in CMS-HL rice. **  A Antibody labeling of the mitochondria at the premeiotic meiocytes stage in YtA anthers. The mitochondria have become amorphous, and the label mainly accumulates in the mitochondria. Bar=200nm. B Mitochondria of the root tip, gold particles are visible. Bar=100nM.C Anthers from YtB at the premeiotic meiocytes stage show zero cross-reactivity. Bar= 100nm. Arrows indicate the location of gold particles. Click here for file

Additional file 3 **Difference between CMS-HL and CMS-BT  A Pollen of CMS-HL (left) and CMS-BT (right) in 1% KI-I2 solution.** Pollen of CMS-HL was unstainable spherical abortive in 1% KI-I2 solution, while pollen of CMS-BT was stainable spherical abortive in 1% KI-I2 solution. B DNA and protein sequence of orfH79 and orf79. There are only five nucleotide variations between the DNA sequences, and lead to changes of five amino acids. Click here for file

Additional file 4 **Western blot analysis of the specificity of the antiserum using ORFH79 expressed in E.coli  A SDS-PAGE analysis of orfH79 expression in E.coli. B Western blot profile of ORFH79 protein in E.coli using the antiserum.**  P5x-2: prokaryotic expression vector pGEX5x-2, p5x-2-H79: recombinant plasmid;  (+), with 1mM IPTG, (-), without IPTG. Black arrow indicated the recombinant protein (GST+ORFH79) and the white arrow indicated the tag (GST) protein Click here for file
