# Supplementary figures and images for: The mitochondrial gene orfH79 plays a critical role in impairing both male gametophyte development and root growth in CMS-Honglian rice
Source: BMC Plant Biol. 2010 Jun 24;10:125. doi: 10.1186/1471-2229-10-125 (PMC3017818; doi:10.1186/1471-2229-10-125)

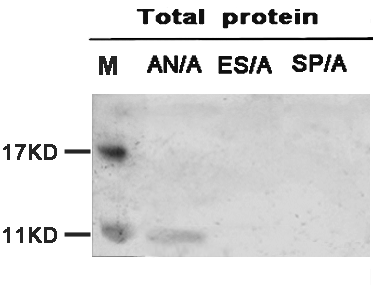

Supplement: Additional file 1 — Western blot analysis of the total protein extracted from anthers, etiolated shoots, and spikelet without mitochondria purification in the YtA line. AN: anthers, ES: etiolated shoots, SP: spikelet [file 1471-2229-10-125-S1.TIFF]

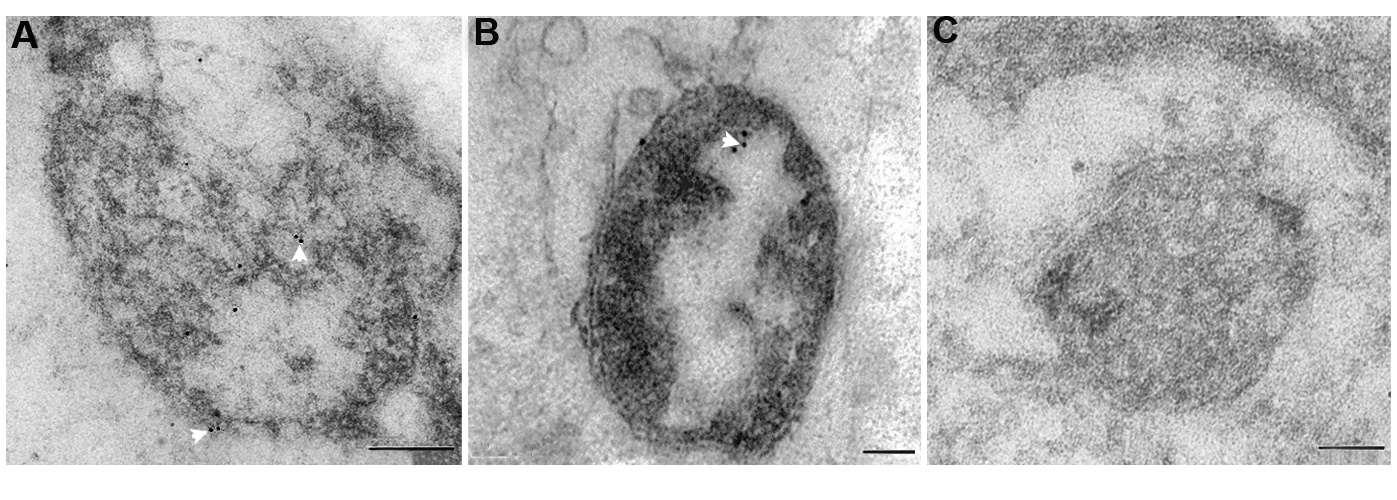

Supplement: Additional file 2 — Immunogold localization of ORFH79 in CMS-HL rice. A Antibody labeling of the mitochondria at the premeiotic meiocytes stage in YtA anthers. The mitochondria have become amorphous, and the label mainly accumulates in the mitochondria. Bar=200nm. B Mitochondria of the root tip, gold particles are visible. Bar=100nM.C Anthers from YtB at the premeiotic meiocytes stage show zero cross-reactivity. Bar= 100nm. Arrows indicate the location of gold particles. [file 1471-2229-10-125-S2.TIFF]

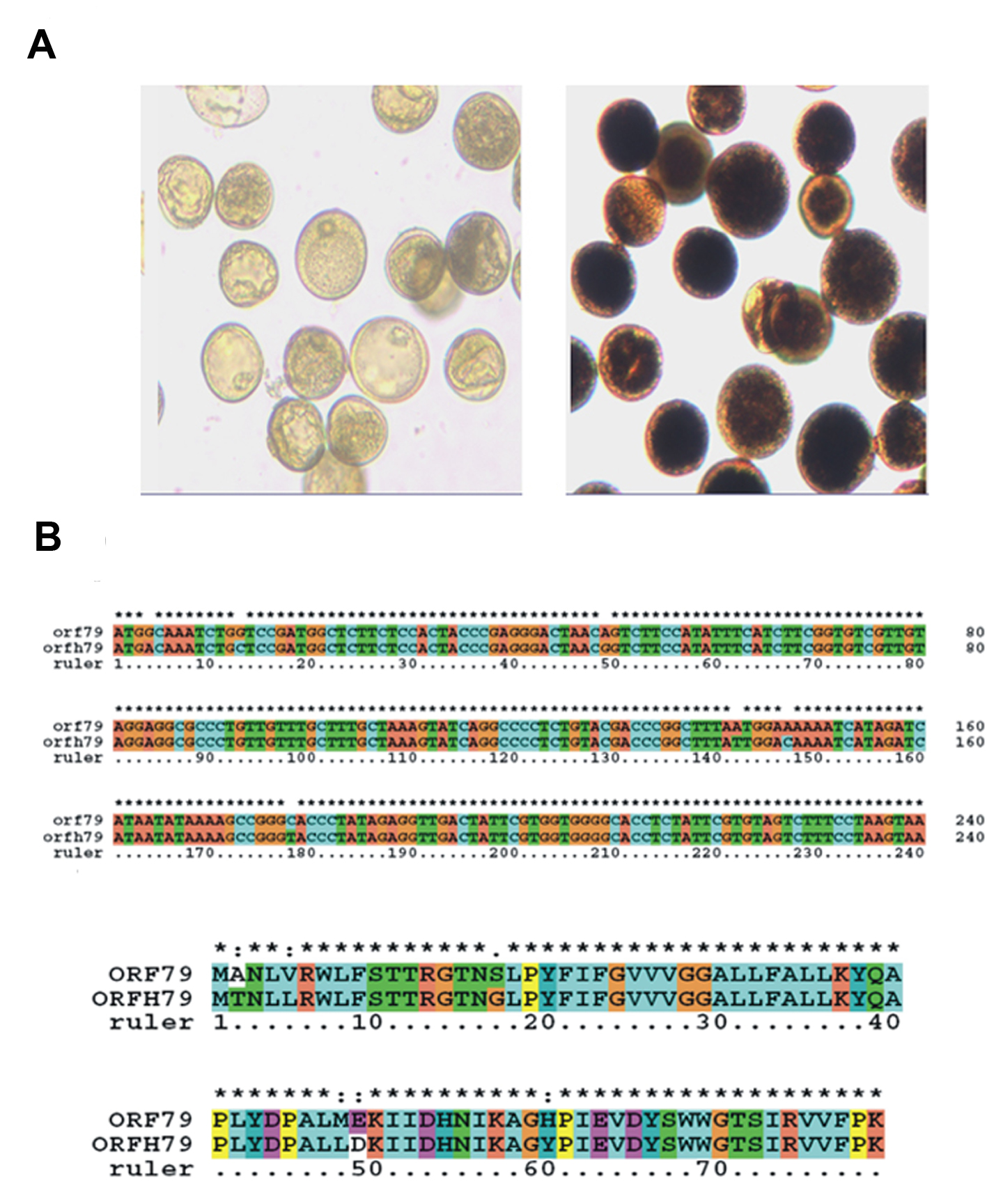

Supplement: Additional file 3 — Difference between CMS-HL and CMS-BT A Pollen of CMS-HL (left) and CMS-BT (right) in 1% KI-I2 solution. Pollen of CMS-HL was unstainable spherical abortive in 1% KI-I2 solution, while pollen of CMS-BT was stainable spherical abortive in 1% KI-I2 solution. B DNA and protein sequence of orfH79 and orf79. There are only five nucleotide variations between the DNA sequences, and lead to changes of five amino acids. [file 1471-2229-10-125-S3.JPEG]

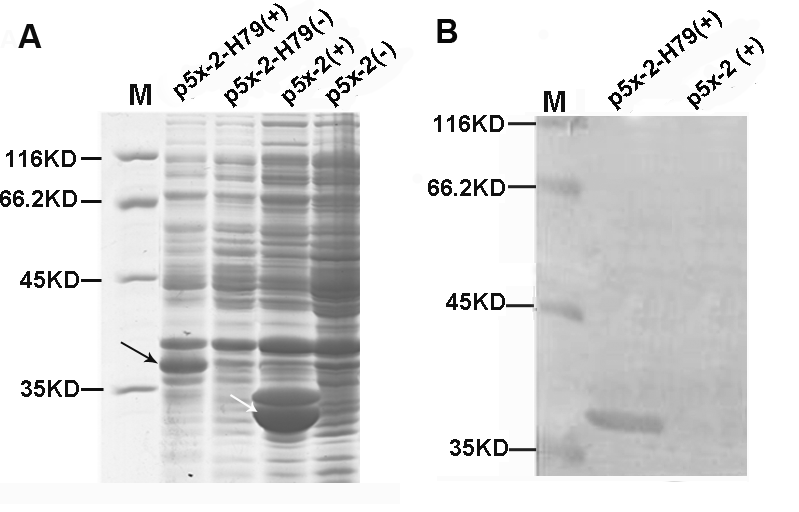

Supplement: Additional file 4 — Western blot analysis of the specificity of the antiserum using ORFH79 expressed in E.coli A SDS-PAGE analysis of orfH79 expression in E.coli. B Western blot profile of ORFH79 protein in E.coli using the antiserum. P5x-2: prokaryotic expression vector pGEX5x-2, p5x-2-H79: recombinant plasmid; (+), with 1mM IPTG, (-), without IPTG. Black arrow indicated the recombinant protein (GST+ORFH79) and the white arrow indicated the tag (GST) protein [file 1471-2229-10-125-S4.TIFF]
